# A pooled analysis of the side effects of non-invasive Transcutaneous Auricular Vagus Nerve Stimulation (taVNS)

**DOI:** 10.3389/fnhum.2025.1539416

**Published:** 2025-02-05

**Authors:** Manon Giraudier, Carlos Ventura-Bort, Christoph Szeska, Mathias Weymar

**Affiliations:** ^1^Department of Biological Psychology and Affective Science, Faculty of Human Sciences, University of Potsdam, Potsdam, Germany; ^2^Faculty of Health Sciences Brandenburg, University of Potsdam, Potsdam, Germany

**Keywords:** side effects, tVNS, taVNS, safety profile, pooled data

## Abstract

**Introduction:**

Transcutaneous auricular vagus nerve stimulation (taVNS) is a promising technique for modulating vagal afferent fibers non-invasively and has shown therapeutic potential in neurological, cognitive, and affective disorders. While previous research highlights its efficacy, the safety profile of taVNS has been less extensively examined.

**Methods:**

This study therefore aimed to systematically investigate side effects of taVNS in a large pooled dataset consisting of *n* = 488 participants, utilizing a standardized questionnaire to assess ten reported side effects. Analyses included effects of stimulation type (interval vs. continuous), stimulation duration, stimulation intensity and participant characteristics (age and gender) as potential modulators.

**Results:**

The findings support the safety of taVNS, with minimal and mild side effects reported across participants (*M* = 1.86, *SD* = 1.36). Although participants receiving sham stimulation were 32.4% less likely to report unpleasant feelings compared to participants receiving taVNS, this effect was driven primarily by low-end ratings (specifically, a rating of 1, indicating *not at all* when experiencing the corresponding side effect), thus suggesting limited clinical relevance. Interval stimulation notably reduced the likelihood of some side effects, particularly for neck pain, dizziness and unpleasant feelings, suggesting potential for optimizing taVNS protocols. Stimulation intensity and duration showed few statistically significant, but clinically minimal (i.e., very small) effects.

**Conclusion:**

Overall, these findings demonstrate a favorable safety profile of taVNS, with mostly mild and transient effects, supporting its use as a suitable non-invasive tool in both research and clinical applications.

## 1 Introduction

The vagal afferent system constitutes a broad network of sensory neural fibers that serve as primary routes by which the brain receives information about bodily processes. These vagal fibers connect to central brain regions including noradrenergic, GABAergic and cholinergic transmission (Yuan and Silberstein, 2016). This anatomical peculiarity has inspired numerous studies to test whether a stimulation of the vagus nerve may thus be a useful adjunct to the treatment of disorders, that are associated with a dysregulation in these transmitter systems, including neurological (e.g., epilepsy), neurocognitive (e.g., mild cognitive impairment) as well as affective and anxiety disorders (for review see Groves and Brown, 2005). Historically, invasive techniques such as implanted vagus nerve stimulation (VNS) were commonly used, despite their risks and potential side effects (Révész et al., 2016). However, with advancements in technology and patient care, there is increasing interest in non-invasive methods that offer greater safety, reduced risks, and improved tolerability. By stimulating the vagus nerve through the ear on the cymba conchae—an area exclusively innervated by the auricular branch of the vagus nerve (Ellrich, 2011), transcutaneous auricular vagus nerve stimulation (taVNS) presents a promising non-invasive alternative to invasive VNS. This offers the potential to provide similar therapeutic benefits without the need for invasive surgery or its associated complications (Ben-Menachem et al., 2015). Indeed, taVNS has been shown to provide benefits, similar to VNS, for the treatment of a wide range of clinical disorders, such as chronic pain (Napadow et al., 2012), depression (Fang et al., 2016) and pharmacoresistant epilepsy (Rong et al., 2014; Aihua et al., 2014; Bauer et al., 2016). Furthermore, taVNS has also shown promising results on various psychological processes in healthy individuals, including cognitive control (e.g., Rufener et al., 2018; Fischer et al., 2018; Maraver et al., 2020), learning and memory (e.g., Jacobs et al., 2015; Szeska et al., 2020; Giraudier et al., 2020; Ventura-Bort et al., 2021; Ventura-Bort and Weymar, 2024), interoception (e.g., Villani et al., 2019; Richter et al., 2021; Ventura-Bort and Weymar, 2024) and motivation (e.g., Neuser et al., 2020).

In light of the promising effects of taVNS, recent studies have focused on identifying the optimal stimulation parameters to enhance its efficacy. As researchers continue to fine-tune these stimulation parameters to enhance the therapeutic and cognitive benefits of taVNS, it is crucial to ensure that these optimizations do not compromise safety. Thus, understanding the safety profile of taVNS is essential to better inform its application in both clinical and research settings. Previous studies have identified some side effects of taVNS, such as skin irritation (Lampros et al., 2021; Evensen et al., 2022; Ventura-Bort and Weymar, 2024), headaches (Lampros et al., 2021), pain (Mertens et al., 2020) and dizziness (Aihua et al., 2014; Jacobs et al., 2015), which, although generally mild and transient, may be linked to the anatomical features of the vagal afferent network. For example, skin irritation, neck contractions and neck pain may result from the stimulation of somatic afferent fibers within the auricular branch of the vagus nerve (ABVN) (Ruffoli et al., 2011), which innervates the external ear canal as well as parts of the larynx, pharynx and proximal esophagus (Jackson, 1949; Panebianco et al., 2016; Oliveira et al., 2017; Möbius and Welkoborsky, 2022) (for review see Yuan and Silberstein, 2016). Electrical stimulation in these areas might trigger localized responses, such as increased blood flow and sensitivity, as well as muscle contractions in the neck, potentially causing symptoms and discomfort. On the other hand, headaches and dizziness could be linked to the activation of the nucleus tractus solitarius (NTS) and its projections the locus coeruleus (LC) (Yakunina et al., 2017) (for review see Henssen et al., 2019). These regions are known to regulate autonomic functions such as blood pressure and heart rate, which could be disrupted during vagal nerve stimulation (Guiraud et al., 2016; Yuan and Silberstein, 2016). However, many studies do not report significant side effects (Kim et al., 2022) and so far only two studies have systematically examined the safety and tolerability of taVNS by comparing multiple studies. Redgrave et al. (2018) concluded that taVNS is generally well-tolerated with mild side effects like skin irritation, headaches, and nasopharyngitis. However, their review included a broader range of transcutaneous VNS techniques, making it difficult to isolate taVNS-specific side effects. More recently, Kim et al. (2022) conducted a systematic review and meta-analysis focused exclusively on taVNS and found that the most commonly reported side effects were ear and headache pain, tingling, and skin redness. Notably, these effects were generally mild and transient. Their analysis also explored the relationship between stimulation parameters and the likelihood of side effects. While they found no significant association between age or gender and the development of side effects, they did identify a positive significant relationship between stimulation duration and the likelihood of side effects. However, variability across the included studies was high (e.g., different stimulation devices and side effect reporting methods).

Although these systematic reviews and meta-analyses highlight the generally mild and transient nature of taVNS side effects, it remains important to further clarify how specific stimulation parameters might influence the occurrence and severity of these effects. The present study therefore aimed to investigate potential side effects of taVNS by pooling together raw data from multiple studies of our own lab. Through a cumulative link mixed model approach, we explored how stimulation parameters, such as stimulation type (continuous stimulation vs. interval stimulation), stimulation intensity and the duration of stimulation, as well as age and gender may influence the occurrence and severity of potential side effects. To address previous limitations (e.g., high variability in study characteristics), only studies were included that employed the same taVNS device with the stimulator consistently placed in the same position of the ear for both active taVNS (i.e., cymba cochae) and control stimulation (i.e., earlobe), as well as the same questionnaire and scale to report potential side effects. This questionnaire (adapted from Jacobs et al., 2015) assessed physical symptoms such as headaches, nausea, dizziness, neck pain, muscle contractions in the neck/face, stinging sensations under the electrode, and skin irritation at the ear, as well as psychological symptoms like concentration fluctuations, mood changes, and unpleasant feelings. It was hypothesized that taVNS would be well tolerated and would not lead to increased reports of these symptoms compared to sham stimulation, with no significant differences expected between the two conditions. Ultimately, the goal of this study is to contribute to the development of safer and more effective taVNS protocols.

## 2 Methods

### 2.1 Sample

Side effect ratings were available for a total of 488 healthy participants across ten included studies that applied taVNS and sham control stimulation (*M*_*age*_ = 23.51, *SD*_*age*_ = 4.73, 71.1% female). All studies were conducted either at the University of Greifswald or at the University of Potsdam, with informed written consent given by all participants. All participants were German speakers (at least C1 level) and had normal or corrected-to-normal vision. Exclusion criteria were neurological or psychiatric disorders, brain surgery, undergoing medication or drug use, pregnancy, a history of migraine and/or epilepsy, cardiac diseases, metal pieces in the body (i.e., a pacemaker), and active implants or physical alterations in the ear (e.g., a cochlear implant). Ethical approval for the study protocols was obtained in accordance with the principles outlined in the Declaration of Helsinki.

Detailed characteristics of each study and participants demographics are provided in Table 1. Additional information regarding data collection for published studies can be found in their respective publications. All raw data from this study have been made publicly available on the Open Science Framework (OSF) platform (https://osf.io/cdvkr/).

**Table 1:** Overview of the study characteristics and the stimulation parameters.

**Study**	**Reference**	**N**	**Task**	**Design**	**Stimulation duration**	**Stimulation type**
1	Ventura-Bort et al. (2018)	*N = 21, *M*_*age*_ = 20.3*	Oddball paradigm	Within-subject	35 min	Continuous
2	Giraudier et al. (2020)	*N* = 61, *M*_*age*_ = 23.4	Lexical decision	Between-subject	23 min	Interval
3	Ventura-Bort et al. (2021)	*N = 36, *M*_*age*_ = 23.1*	Passive viewing	Within-subject	7 min	Continuous
4	Incoronato et al. (2021)^1^	*N = 67, *M*_*age*_ = 23.5*	Passive viewing	Between-subject	46 min	Interval
5	Ventura-Bort and Weymar, (2024)^2^	*N = 27, *M*_*age*_ = 23.8*	N-back paradigm	Within-subject	40 min	Interval
6	Ventura-Bort et al. (2024)	*N = 31, *M*_*age*_ = 21.3*	Passive viewing	Within-subject	15 min	Interval
7	Ventura-Bort et al. (2024)	*N = 65, *M*_*age*_ = 24.3*	Passive viewing	Between-subject	15 min	Interval
8	Giraudier and Weymar (2024)^3^	*N = 57, *M*_*age*_ = 24.0*	Passive viewing	Within-subject	35 min	Interval
9	Ventura-Bort and Weymar (2024)	*N = 53, *M*_*age*_ = 23.8*	Heart beating counting	Within-subject	45 min	Interval
10	Giraudier et al. (2024)	*N = 70, *M*_*age*_ = 23.9*	Serial reaction time, oddball paradigm	Within-subject	80 min	Continuous, interval

Oddball task: participants completed a rotated-heads task with standards, targets, and novel stimuli to assess task performance. Lexical decision task: participants determined whether letter strings were real German words, focusing on speed and accuracy. Passive viewing task: participants passively viewed emotional or neutral images. N-back task: a working memory task requiring participants to match current stimuli to those presented previously. Heart-beating counting: participants focused on counting their own heartbeats, providing a measure of interoceptive awareness. Serial reaction time task: participants responded to sequences of visual stimuli by pressing corresponding keys, examining response selection and sequence learning. The studies employed both within- and between-subject designs, with stimulation durations ranging from 7 to 80 min and stimulation types being either continuous, interval (alternating in 30-s cycles of on and off phases), or both.

### 2.2 Transcutaneous auricular vagus nerve stimulation

The device that was used for stimulation in all the studies consisted of two titanium electrodes mounted on a holder resembling in-ear headphones, connected to a battery-operated stimulation unit (CMO2, Cerbomed, Erlangen, Germany). In the taVNS condition, electrodes were positioned below the tragus in the cymba concha area, which has been shown to have the densest projections of the auricular branch of the vagus nerve (ABVN) (Badran et al., 2018; Peuker and Filler, 2002). In the sham condition, electrodes were attached to the center of the earlobe, an area assumed to lack vagal innervation (Peuker and Filler, 2002). Participants were randomly assigned to receive either taVNS or sham stimulation using predetermined randomization lists across all studies, ensuring objective allocation. Electrical stimulation was delivered either continuously or in intervals alternating in 30-s cycles of on and off phases. The preset parameters of the device included a pulse width of 250 μs and a frequency of 25 Hz. The included studies employed a single-blind design, where participants were blinded to the stimulation condition, but experimenters were necessarily aware due to device setup requirements. The stimulation intensity was individually calibrated in all the included studies using the same standardized procedure. Participants underwent a series of stimulation trials and rated their subjective sensation of the stimulation on a 11-point scale, ranging from 1 (nothing), 3 (light tingling), 6 (strong tingling) to 10 (painful). Stimulation began at an intensity of 0.1 mA, which was incrementally increased by 0.1 mA until participants reported a sensation of 9. This intensity was repeated to confirm the rating before gradually decreasing in 0.1 mA steps until participants reported a sensation of 6 or below. This process was conducted twice, and the final stimulation intensity was calculated as the average of the four intensities rated as 8 (two from the increasing and two from the decreasing trials). This calibration ensured the intensity was above the sensory threshold but below the pain threshold, accounting for individual differences in sensitivity while maintaining participant comfort. The mean stimulation intensity for the taVNS condition was 1.35 mA (range: 0.1–4.6 mA) and for the sham condition, it was 1.52 mA (range: 0.1–4.5 mA).

### 2.3 Side effects

At the end of each experiment, all participants completed a questionnaire (adapted from Jacobs et al., 2015) to assess potential side effects of the stimulation on a 7-point scale (1 being *not at all* and 7 being *very much*). The assessed potential side effects included physical symptoms such as headaches, nausea, dizziness, neck pain, muscle contractions in the neck/face, stinging sensations under the electrode, and skin irritation at the ear, as well as psychological symptoms like concentration fluctuations, mood changes, and unpleasant feelings.

### 2.4 Statistics

All statistical analyses were carried out in the R environment (R Core Team, 2023). To test whether the reported severity of the side effects differed between taVNS and sham stimulation conditions and to explore whether additional predictors modulate the relationship between taVNS and the reported side effects, we conducted a series of cumulative link mixed models (CLMMs) for each side effect using the ordinal package (Christensen, 2019). As fixed effects, we included the effect of *stimulation* (taVNS vs. sham), the effect of *stimulation type* (interval vs. continuous stimulation), the effect of *gender* (female vs. male vs. diverse), and the effects of *stimulation intensity, stimulation duration* and *age* (group mean-centered; see Richter and Naumann, 2002). As random factors, we included *participant* (*N* = 429) and *study* (*N* = 9) with a total amount of 665 observations (i.e., due to missing data in Ventura-Bort et al., 2018). We included a random intercept for each participant, with participants nested within their respective studies. The selected random-effect structure was identical for all models and included theoretically relevant variance components (c.f., Bates et al., 2015).

To determine the best-fitting model for each side effect, pairwise model comparisons were performed using likelihood ratio tests and the Akaike Information Criterion (AIC) (Meteyard and Davies, 2020). Each comparison evaluated whether the inclusion of interaction effects between key predictors improved model fit compared to the full model with only main effects. For example, to test whether the interaction between stimulation intensity and stimulation duration improved the model fit for headaches, we compared the full model with the model containing the interaction term: If the model with the interaction term showed a significantly better fit (e.g., a smaller AIC, or a significant *p*-value from the likelihood ratio test), it was retained as the best model. This systematic approach was conducted separately for each side effect and ensured that the final models for each side effect were optimally specified, allowing us to identify significant predictors and interactions while controlling for participant- and study-level variability. The models were fit using Maximum Likelihood (ML) estimation, as this method is preferred over restricted ML (REML) when comparing models with different fixed effects (Bates et al., 2015; Meteyard and Davies, 2020).

To interpret the effect sizes in each model, we reported the odds ratio (OR) for the predictors (McGough and Faraone, 2009). The OR represents the odds of an outcome occuring given a particular exposure compared to its absense (Szumilas, 2010) and provides an intuitive measure of effect strength. An OR of 1 implies that the exposure (i.e., taVNS) does not affect the likelihood of the outcome (i.e., the side effect) compared to the reference group (i.e., sham stimulation).

## 3 Results

### 3.1 Descriptive data

**Figure 1:**
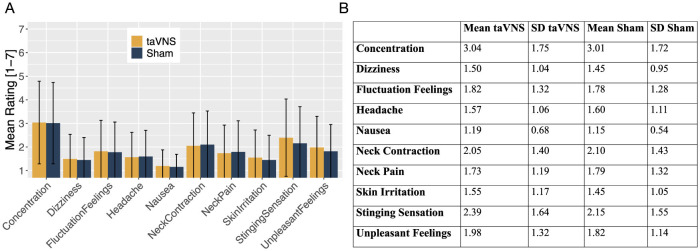
**(A)** Mean ratings of all the assessed side effects for taVNS (orange) and sham stimulation (blue), presented as bar charts. **(B)** Table displaying the mean ratings and standard deviations (SD) for all assessed side effects, comparing taVNS and sham stimulation.

The descriptive data indicate that the ratings of all assessed side effects were generally low on average. As shown in Figure 1, the subjective ratings were on average below 2 (*M* = 1.86, *SD* = 1.36), suggesting that participants experienced minimal side effects overall.

### 3.2 Effects of stimulation

**Figure 2:**
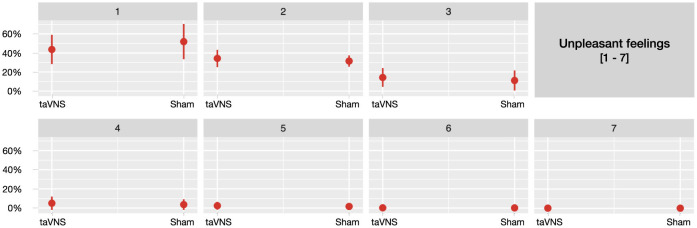
Predicted probabilities of the side effect *unpleasant feelings* for taVNS and sham stimulation, separately across seven plots corresponding to each point on the 7-point scale (1 being *not at all* and 7 being *very much*).

Stimulation did not result in any significant differences for the majority of the assessed side effects, including headaches, neck pain, neck contractions, dizziness, skin irritation, concentration and fluctuations in feelings (*ps*>0.07). Detailed results for non-significant effects can be found in the Appendices A1–A10. However, participants in the sham group were 32.4% less likely to report unpleasant feelings compared to those who received taVNS (β = −0.39, *p* = 0.040;*OR* = 0.676). Notably, however, this effect was driven primarily by ratings in the lowest range of the scale for unpleasant feelings, as illustrated in Figure 2.

### 3.3 Effects of stimulation type

The type of stimulation played a noteworthy role in moderating several side effects. Compared to continuous stimulation, interval stimulation was associated with a 51.6% reduction in the likelihood of reporting neck pain (β = −0.73, *p* = 0.042;*OR* = 0.484). Furthermore, interval stimulation combined with higher stimulation intensity further reduced dizziness (β = −0.78, *p* = 0.047;*OR* = 0.460) and unpleasant feelings (β = −0.79, *p* = 0.040;*OR* = 0.454), both reflecting a 54% reduction in likelihood. Participants receiving sham interval stimulation reported significantly lower levels of nausea (β = −2.39, *p* = 0.018;*OR* = 0.092), corresponding to a 91% reduction, and lower levels of stinging sensations (β = −0.93, *p* = 0.038;*OR* = 0.396), corresponding to a 60% reduction, compared to other participants.

### 3.4 Effects of stimulation duration

Longer stimulation durations were associated with an ~6% increase in the odds of experiencing several side effects, including headaches (β = 0.03, *p* < 0.001;*OR* = 1.027), neck pain (β = 0.06, *p* < 0.001;*OR* = 1.059), neck contractions (β = 0.06, *p* < 0.001;*OR* = 1.059) and concentration issues (β = 0.06, *p* < 0.001;*OR* = 1.060). Moreover, stimulation duration interacted with stimulation type for some side effects. The effect of longer stimulation durations was mitigated for interval stimulation compared to continuous stimulation for both neck pain (β = −0.03, *p* = 0.013;*OR* = 0.972) and neck contractions (β = −0.03, *p* = 0.004;*OR* = 0.966), with an ~3% decrease in the odds of experiencing these side effects. For concentration, the positive effect of longer stimulation was reduced during interval stimulation (β = −0.04, *p* = 0.003;*OR* = 0.958). Specifically, for participants receiving interval stimulation, longer stimulation durations were associated with a slightly lower probability (4.2% lower odds) of reporting concentration issues (OR = 0.958).

### 3.5 Effects of stimulation intensity

Higher stimulation intensity was associated with a reduced likelihood of experiencing neck contractions (β = −0.33, *p* = 0.036;*OR* = 0.721), stinging sensations (β = −0.39, *p* = 0.002;*OR* = 0.674), skin irritation (β = −0.67, *p* = 0.028;*OR* = 0.510) and fluctuactions in feelings (β = −0.42, *p* = 0.040;*OR* = 0.659). During interval stimulation, higher stimulation intensity was further associated with less unpleasant feelings (β = −0.79, *p* = 0.040;*OR* = 0.454) and decreased dizziness ratings (β = −0.78, *p* = 0.047;*OR* = 0.460).

### 3.6 Effects of age and gender

Age showed a notable effect on concentration. The negative coefficient indicates an inverse relationship between age and the likelihood of experiencing concentration-related side effects (β = −0.06, *p* = 0.038;*OR* = 0.943), implying that older participants were associated with lower levels of concentration issues.

Males were significantly less likely to report severe headaches compared to females (β = −1.06, *p* = 0.008;*OR* = 0.347) and were also less likely to experience unpleasant feelings (β = −0.89, *p* = 0.008;*OR* = 0.412).

## 4 Discussion

Previous work has suggested that taVNS can lead to mild and transient side effects compared to sham stimulation, although findings across individual studies have been inconsistent. Some studies report side effects such as skin irritation (Fischer et al., 2018; Lampros et al., 2021; Evensen et al., 2022; Ventura-Bort and Weymar, 2024), headaches (Lampros et al., 2021), pain (Mertens et al., 2020) and dizziness (Aihua et al., 2014; Jacobs et al., 2015). Others, however, do not find evidence of side effects (Busch et al., 2013; Giraudier et al., 2020; Ricci et al., 2020; Sharon et al., 2021; Konjusha et al., 2023), and many studies do not even report on side effects at all (cf. Kim et al., 2022). To reduce variability and address inconsistencies found in previous research (cf. Kim et al., 2022), the present project aimed to clarify the safety profile of taVNS by systematically examining side effects across a large and more homogeneous dataset, for instance by only including studies using the same taVNS device, stimulation position and standardized side effect questionnaire.

Overall, participants reported low ratings for all assessed side effects and taVNS was generally not associated with a higher severity of side effects compared to sham stimulation, supporting our hypothesis. However, participants receiving sham stimulation were 32.4% less likely to report unpleasant feelings compared to those receiving taVNS. Notably, this effect was primarily driven by a higher likelihood of sham participants selecting a rating of 1 (indicating *not at all* when experiencing unpleasant feelings). In contrast, participants receiving taVNS were less likely to report this lowest rating. Importantly, as ratings increased beyond 1, the probabilities remained comparable across the higher ratings (2 through 7). This indicates that the observed difference was limited to the lowest range of unpleasant feelings and did not persist at higher levels, suggesting that the practical relevance of this effect is limited and can be dismissed. Moreover, participants receiving interval stimulation, compared to those receiving continuous stimulation, demonstrated an ~54% reduction in the likelihood of reporting neck pain, dizziness and unpleasant feelings (for dizziness and unpleasant feelings particularly in combination with higher stimulation intensities). This advantage of interval stimulation in reducing the likelihood of some of the assessed side effects independently of vagal activation may be attributed to the recovery periods it offers, which could help alleviate discomfort that continuous stimulation tends to provoke. Alternatively, this effect could also be attributed to differences in total gate charge, as interval stimulation consists of on and off phases, thereby reducing the total gate charge compared to continuous stimulation. Surprisingly, interval stimulation was associated with fewer side effects, specifically a reduced likelihood of reporting nausea and stinging sensations, particularly among participants receiving sham stimulation. However, these differences were most pronounced at the lowest rating levels (i.e., a rating of 1), similarly to the effect of stimulation on unpleasant feelings. One might argue that continuous stimulation, in contrast to interval stimulation, may more consistently activate autonomic centers in the brainstem, such as the NTS and LC (Yakunina et al., 2017), which regulate essential functions like blood pressure and heart rate (for review see Yuan and Silberstein, 2016). While this could theoretically contribute to side effects like nausea and dizziness (i.e., disruptions in these autonomic processes may contribute to the onset of such side effects) (Guiraud et al., 2016), the data does not suggest a clear increase in discomfort with continuous stimulation compared to (sham) interval stimulation. Instead, sham stimulation appears to be associated with a higher likelihood of participants selecting *not at all* when reporting certain side effects, particularly under interval stimulation. However, since the overall side effect ratings remain low across all stimulation conditions and types, this suggests that taVNS does not significantly increase side effects compared to sham, but rather that sham participants tend to report no side effects more frequently, particularly during interval stimulation.

Although several statistically significant effects were observed for stimulation duration, the magnitude of these effects suggested limited clinical relevance. Specifically, longer stimulation was associated with an increased likelihood of experiencing headaches, neck discomfort and concentration issues. However, the small coefficients (ranging from 0.03 to 0.06) and odds ratios (close to 1) indicate minimal changes in the likelihood of experiencing these side effects, even with extended stimulation periods. This implies that while these effects were statistically significant, they likely do not represent a meaningful clinical impact (Deyo and Patrick, 1995). Similarly, the effect of interval stimulation, which combined with longer durations was associated with a reduced likelihood of reporting neck discomfort, also exhibited small coefficients and odd ratios. In the case of concentration, while longer durations during interval stimulation were associated with a reduced likelihood of concentration issues, the small coefficient suggests that the effect is minimal. This underscores the importance of not relying solely on *p*-values for interpreting the significance of findings; instead, focusing on the coefficients provides a clearer understanding of the practical implications of these findings (Solla et al., 2018; Halsey, 2019). Nonetheless, it is also important to note that these effects may be more related to the overall duration of the experimental task rather than taVNS itself (i.e., which might have led to general discomfort or fatigue, potentially contributing to headaches, neck discomfort and concentration difficulties independent of the stimulation) (Ackerman and Kanfer, 2009). In practical applications, reducing task duration or incorporating breaks could help minimize the likelihood of fatigue-related side effects.

Equally relevant for the setup of taVNS studies is the calibration of the stimulation intensity, which was adjusted individually for each participant in all included studies so that it was above the sensory threshold but below the pain threshold. This approach, while ensuring participant comfort, also introduces variability based on individual sensitivity levels. Participants who tolerated higher intensities possibly had a naturally higher threshold for discomfort, influencing not only their chosen stimulation intensity but also their lower reports of side effects such as neck contractions, stinging sensations, fluctuations in feelings and unpleasant feelings. Indeed, previous research showed that individual sensitivity levels significantly influence side effect reports and symptom attribution (Petrie et al., 2004; Faasse et al., 2015; MacKrill et al., 2021). This suggests that the reduced likelihood of these side effects at higher intensities may be more related to individual pain tolerance rather than intensity itself. While individually calibrated stimulation offers some advantages (e.g., same amount of distraction due to the stimulation), it may also introduce biases by effectively grouping participants based on their sensitivity levels, highlighting the importance of further investigating individual differences in sensitivity and their potential influence on side effects of taVNS. Similarly, individual differences across participants were also evident in the effects of age and gender on side effects, contrasting previous results (Kim et al., 2022). Older participants in the present dataset reported lower levels of concentration difficulties independent of stimulation, which contrasts with the expectation that older individuals may be more vulnerable to treatment-induced side effects, particularly cognitive impairments (e.g., Magnuson et al., 2016). However, older participants in this dataset consisted entirely of students and were not representative of elderly individuals (i.e., as the maximum age was 46 years). This effect aligns with existing literature indicating that pain thresholds tend to increase with age (Lautenbacher et al., 2017), potentially explaining the lower reports of discomfort among older participants. However, the statistical significance of this effect should be interpreted with caution, as the coefficient of –0.06 reflects only minimal changes in likelihood, suggesting limited clinical relevance. Moreover, male students were found to be significantly less likely to report severe headaches compared to female students and they were also less likely to experience unpleasant feelings. This, however, aligns with findings showing gender differences in experiencing and reporting adverse effects, suggesting that females may be more sensitive or more likely to report discomfort in response to various treatments (e.g., Jokerst et al., 1999; Haack et al., 2009; Alghamdi et al., 2021; Colombo et al., 2016).

While our pooled analysis offers valuable insights, several limitations must be acknowledged. First, there is noticeable heterogeneity across studies, for instance with diverse cognitive tasks (see Table 1), potentially affecting the reported side effects. Additionally, variability in stimulation protocols, such as the limited use of continuous stimulation (applied in only three studies) compared to interval stimulation, may introduce biases and affect the reliability of the findings. The broad range of stimulation durations (7–80 min) across studies could have further contributed to variability in outcomes. Addressing these limitations in future research is crucial, for example, including a more balanced representation of stimulation types and establishing standardized protocols would minimize variability, improve comparability across studies, and provide clearer insights into the effects of taVNS parameters on side effects. Moreover, the current study focused solely on subjective ratings to assess side effects. Future research could also consider incorporating physiological assessments to further enhance our understanding of the safety profile of taVNS. Despite these limitations, the consistently low levels of reported side effects support the general safety and tolerability of taVNS, emphasizing its potential as a safe intervention and informing future protocol optimization.

In summary, our findings support the hypothesis that taVNS does not lead to increased adverse side effects compared to sham stimulation, especially with the moderating effects of stimulation type (interval stimulation). Importantly, the low levels of reported side effects across participants suggests that, if present, they are relatively mild and manageable. While our results revealed statistically significant effects of stimulation type, duration, and intensity on side effects, it is important to consider whether these findings are clinically relevant. Some effects, such as the influence of interval stimulation on neck pain, dizziness, and unpleasant feelings, may hold potential for optimizing taVNS protocols. However, the magnitude of certain effects, such as the impact of longer stimulation durations, requires further consideration of their practical significance. Our results show a favorable safety profile for taVNS, with most side effects being very mild and well-tolerated, supporting taVNS as a safe, non-invasive tool in basic and clinical research studies.

## Data Availability

The datasets presented in the study can be found in the following online repository: https://osf.io/cdvkr/.

## Footnotes

^1^Incoronato, G., Giraudier, M., Ventura-Bort, C., and Weymar, M. (2021). Effects of transcutaneous vagus nerve stimulation (tVNS) on episodic memory for trustworthy and untrustworthy faces: an ERP study [unpublished data].

^2^Ventura-Bort, C., and Weymar, M. (2024). Effects of transcutaneous auricular vagus nerve stimulation (taVNS) on working memory [unpublished data].

^3^Giraudier, M., and Weymar, M. (2024). Effects of transcutaneous auricular vagus nerve stimulation (taVNS) on the processing and recognition of emotional and neutral faces [unpublished data].
