# Supplementary material for: A pooled analysis of the side effects of non-invasive Transcutaneous Auricular Vagus Nerve Stimulation (taVNS)
Source: Front Hum Neurosci. 2025 Feb 5;19:1539416. doi: 10.3389/fnhum.2025.1539416 (PMC11841445; doi:10.3389/fnhum.2025.1539416)
Supplement: Supplementary file 1 [file Table_1.docx]

|  |  | Fixed Effects | | |
| --- | --- | --- | --- | --- |
| Predictor | **Est** | **SE** | **z** | **p** |
| Stimulation intensity | -0.16 | 0.18 | -0.91 | 0.366 |
| Stimulation duration | 0.03 | 0.01 | 3.76 | **< 0.001**** |
| Stimulation (sham-taVNS) | -0.05 | 0.22 | -0.21 | 0.834 |
| Stimulation type (interval-continuous) | -0.06 | 0.40 | -0.14 | 0.886 |
| Age | -0.04 | 0.03 | -1.24 | 0.215 |
| Gender (male-female) | -1.06 | 0.40 | -2.67 | **0.008**** |
| Gender (diverse-female) | 0.43 | 1.51 | 0.29 | 0.773 |
| Stimulation intensity x Stimulation duration | 0.02 | 0.01 | 2.61 | **0.009**** |
|  |  | **Random Effects** | | |
|  |  | **Variance** | | **S.D.** |
| VP:Study (Intercept) |  | 3.996 |  | 1.999 |
| Study (Intercept) |  | 0.000 |  | 0.000 |

**Appendix A1. Model output for the side effect ‘headaches’.**

|  |  | Fixed Effects | | |
| --- | --- | --- | --- | --- |
| Predictor | **Est** | **SE** | **z** | **p** |
| Stimulation intensity | 0.25 | 0.35 | 0.72 | 0.47 |
| Stimulation type (interval-continuous) | -0.10 | 0.51 | -0.21 | 0.84 |
| Stimulation (sham-taVNS) | -0.15 | 0.21 | -0.71 | 0.48 |
| Stimulation duration | 0.01 | 0.01 | 1.06 | 0.29 |
| Age | -0.04 | 0.03 | -1.56 | 0.12 |
| Gender (male-female) | -0.39 | 0.30 | -1.32 | 0.19 |
| Gender (diverse-female) | 0.68 | 1.19 | 0.57 | 0.57 |
| Stimulation intensity x Stimulation type  (interval-continuous) | -0.78 | 0.39 | -1.99 | **0.05*** |
|  |  | **Random Effects** | | |
|  |  | **Variance** | | **S.D.** |
| VP:Study (Intercept) |  | 1.5274 |  | 1.2359 |
| Study (Intercept) |  | 0.1108 |  | 0.3328 |

**Appendix A2. Model output for the side effect ‘dizziness’.**

|  |  | Fixed Effects | | |
| --- | --- | --- | --- | --- |
| Predictor | **Est** | **SE** | **z** | **p** |
| Stimulation type (interval-continuous) | -0.73 | 0.36 | -2.03 | **0.04*** |
| Stimulation duration | 0.06 | 0.01 | 6.00 | **<0.001***** |
| Stimulation (sham-taVNS) | 0.13 | 0.19 | 0.67 | 0.51 |
| Stimulation intensity | -0.20 | 0.15 | -1.32 | 0.19 |
| Age | -0.06 | 0.03 | -1.94 | 0.05. |
| Gender (male-female) | -0.32 | 0.32 | -1.02 | 0.31 |
| Gender (diverse-female) | 1.28 | 1.36 | 0.94 | 0.35 |
| Stimulation duration x Stimulation type  (interval-continuous) | -0.03 | 0.01 | -2.48 | **0.01*** |
|  |  | **Random Effects** | | |
|  |  | **Variance** | | **S.D.** |
| VP:Study (Intercept) |  | 2.625 |  | 1.620 |
| Study (Intercept) |  | 0.000 |  | 0.000 |

**Appendix A3. Model output for the side effect ‘neck pain’.**

|  |  | Fixed Effects | | |
| --- | --- | --- | --- | --- |
| Predictor | **Est** | **SE** | **z** | **p** |
| Stimulation (sham-taVNS) | 0.720 | 0.817 | 0.882 | 0.378 |
| Stimulation type (interval-continuous) | 0.515 | 1.079 | 0.477 | 0.633 |
| Stimulation intensity | -0.911 | 0.497 | -1.834 | 0.067 . |
| Stimulation duration | 0.008 | 0.016 | 0.463 | 0.644 |
| Age | 0.025 | 0.079 | 0.312 | 0.755 |
| Gender (male-female) | 0.342 | 0.883 | 0.387 | 0.699 |
| Gender (diverse-female) | 1.001 | 3.468 | 0.289 | 0.773 |
| Stimulation (sham-taVNS) x Stimulation type  (interval-continuous) | -2.385 | 1.007 | -2.368 | **0.018 *** |
|  |  | **Random Effects** | | |
|  |  | **Variance** | | **S.D.** |
| VP:Study (Intercept) |  | 74.980 |  | 8.659 |
| Study (Intercept) |  | 0.000 |  | 0.000 |

**Appendix A4. Model output for the side effect ‘nausea’.**

|  |  | Fixed Effects | | |
| --- | --- | --- | --- | --- |
| Predictor | **Est** | **SE** | **z** | **p** |
| Stimulation type (interval-continuous) | -0.604 | 0.364 | -1.659 | 0.097 . |
| Stimulation duration | 0.058 | 0.010 | 5.826 | **<0.001***** |
| Stimulation (sham-taVNS) | 0.212 | 0.183 | 1.160 | 0.246 |
| Stimulation intensity | -0.327 | 0.156 | -2.099 | **0.036 *** |
| Age | -0.047 | 0.030 | -1.564 | 0.118 |
| Gender (male-female) | -0.170 | 0.331 | -0.514 | 0.607 |
| Gender (diverse-female) | 1.870 | 1.338 | 1.398 | 0.162 |
| Stimulation duration x Stimulation type (interval-continuous) | -0.035 | 0.012 | -2.870 | **0.004 **** |
|  |  | **Random Effects** | | |
|  |  | **Variance** | | **S.D.** |
| VP:Study (Intercept) |  | 3.831 |  | 1.957 |
| Study (Intercept) |  | 0.000 |  | 0.000 |

**Appendix A5. Model output for the side effect ‘neck contraction’.**

|  |  | Fixed Effects | | |
| --- | --- | --- | --- | --- |
| Predictor | **Est** | **SE** | **z** | **p** |
| Stimulation (sham-taVNS) | 0.415 | 0.401 | 1.035 | 0.301 |
| Stimulation type (interval-continuous) | 0.555 | 0.558 | 0.993 | 0.321 |
| Stimulation intensity | -0.395 | 0.130 | -3.031 | **0.002 **** |
| Stimulation duration | 0.004 | 0.008 | 0.491 | 0.624 |
| Age | -0.030 | 0.035 | -0.859 | 0.390 |
| Gender (male-female) | -0.020 | 0.239 | -0.083 | 0.934 |
| Gender (diverse-female) | 0.402 | 1.012 | 0.398 | 0.691 |
| Stimulation (sham-taVNS) x Stimulation type (interval-continuous) | -0.927 | 0.446 | -2.079 | **0.038 *** |
|  |  | **Random Effects** | | |
|  |  | **Variance** | | **S.D.** |
| VP:Study (Intercept) |  | 1.316 |  | 1.147 |
| Study (Intercept) |  | 0.188 |  | 0.437 |

**Appendix A6. Model output for the side effect ‘stinging sensation’.**

|  |  | Fixed Effects | | |
| --- | --- | --- | --- | --- |
| Predictor | **Est** | **SE** | **z** | **p** |
| Stimulation (sham-taVNS) | -0.532 | 0.303 | -1.755 | 0.079 . |
| Stimulation intensity | -0.674 | 0.307 | -2.195 | **0.028 *** |
| Stimulation type (interval-continuous) | -0.020 | 0.748 | -0.026 | 0.979 |
| Stimulation duration | 0.008 | 0.012 | 0.656 | 0.512 |
| Age | -0.030 | 0.057 | -0.537 | 0.591 |
| Gender (male-female) | 0.352 | 0.653 | 0.539 | 0.590 |
| Gender (diverse-female) | 6.719 | 4.208 | 1.597 | 0.110 |
|  |  | **Random Effects** | | |
|  |  | **Variance** | | **S.D.** |
| VP:Study (Intercept) |  | 53.09 |  | 7.286 |
| Study (Intercept) |  | 0.000 |  | 0.000 |

**Appendix A7. Model output for the side effect ‘skin irritation’.**

|  |  | Fixed Effects | | |
| --- | --- | --- | --- | --- |
| Predictor | **Est** | **SE** | **z** | **p** |
| Stimulation type (interval-continuous) | 0.944 | 0.498 | 1.897 | 0.058 . |
| Stimulation duration | 0.058 | 0.058 | 0.058 | 0.058 |
| Stimulation (sham-taVNS) | -0.058 | 0.169 | -0.345 | 0.730 |
| Stimulation intensity | -0.233 | 0.145 | -1.604 | 0.109 |
| Age | -0.058 | 0.028 | -2.070 | **0.038 *** |
| Gender (male-female) | 0.399 | 0.314 | 1.269 | 0.205 |
| Gender (diverse-female) | -1.697 | 1.542 | -1.101 | 0.271 |
| Stimulation duration x Stimulation type (interval-continuous) | -0.043 | 0.015 | -2.950 | **0.003 **** |
|  |  | **Random Effects** | | |
|  |  | **Variance** | | **S.D.** |
| VP:Study (Intercept) |  | 4.193 |  | 2.048 |
| Study (Intercept) |  | 0.226 |  | 0.476 |

**Appendix A8. Model output for the side effect ‘concentration’.**

|  |  | Fixed Effects | | |
| --- | --- | --- | --- | --- |
| Predictor | **Est** | **SE** | **z** | **p** |
| Stimulation intensity | -0.417 | 0.203 | -2.051 | **0.040 *** |
| Gender (male-female) | -0.706 | 0.403 | -1.752 | 0.080 . |
| Gender (diverse-female) | -0.435 | 2.192 | -0.198 | 0.843 |
| Stimulation (sham-taVNS) | -0.067 | 0.206 | -0.326 | 0.744 |
| Stimulation type (interval-continuous) | 0.397 | 0.455 | 0.873 | 0.383 |
| Stimulation duration | 0.002 | 0.007 | 0.216 | 0.829 |
| Age | -0.058 | 0.036 | -1.626 | 0.104 |
| Stimulation intensity x Gender  (male-female) | 0.892 | 0.495 | 1.804 | 0.071 . |
| Stimulation intensity x Gender  (diverse-female) | -4.649 | 3.588 | -1.296 | 0.195 |
|  |  | **Random Effects** | | |
|  |  | **Variance** | | **S.D.** |
| VP:Study (Intercept) |  | 5.884 |  | 2.426 |
| Study (Intercept) |  | 0.000 |  | 0.000 |

**Appendix A9. Model output for the side effect ‘fluctuations of feelings’.**

|  |  | Fixed Effects | | |
| --- | --- | --- | --- | --- |
| Predictor | **Est** | **SE** | **z** | **p** |
| Stimulation intensity | 0.303 | 0.339 | 0.896 | 0.370 |
| Stimulation type (interval-continuous) | -0.143 | 0.360 | -0.396 | 0.692 |
| Stimulation (sham-taVNS) | -0.391 | 0.190 | -2.058 | 0.040 * |
| Stimulation duration | -0.006 | 0.006 | -1.068 | 0.285 |
| Age | -0.001 | 0.029 | -0.023 | 0.982 |
| Gender (male-female) | -0.888 | 0.337 | -2.638 | **0.008 **** |
| Gender (diverse-female) | 1.225 | 1.293 | 0.948 | 0.343 |
| Stimulation intensity x Stimulation type  (interval-continuous) | -0.788 | 0.383 | -2.058 | **0.040 *** |
|  |  | **Random Effects** | | |
|  |  | **Variance** | | **S.D.** |
| VP:Study (Intercept) |  | 3.79 |  | 1.947 |
| Study (Intercept) |  | 0.000 |  | 0.000 |

**Appendix A10. Model output for the side effect ‘unpleasant feelings’.**
